# Supplementary material for: The genome sequence of hogweed, Heracleum sphondylium L. (Apiales: Apiaceae)
Source: Wellcome Open Res. 2026 Sep 18;11:700. [Version 1] doi: 10.12688/wellcomeopenres.27848.1 (PMC13731896; doi:10.12688/wellcomeopenres.27848.1)
Supplement: Peer review history

# Reviewer report: Reviewer response for version 1

**DOI:** 10.21956/wellcomeopenres.30645.r177669

**Published:** 2026-11-03

**Authors:** Ruslan Kalendar

**Affiliations:** 1 University of Helsinki, Helsinki, Finland

The authors present a haplotype-resolved genome assembly of hogweed, *Heracleum sphondylium* (specimen drHerSpho1, Roslin, Scotland), generated within the Darwin Tree of Life project from PacBio HiFi (176.33 Gb), Hi-C (314.34 Gb) and leaf RNA-seq data. The haplotype 1 assembly spans 2,028.22 Mb in 408 scaffolds (contig N50 6.08 Mb, scaffold N50 179.91 Mb), with 96.53% of the sequence in 11 chromosomal pseudomolecules, consistent with 2n = 22. Quality metrics are high (QV 66.7; BUSCO C = 98.5%; combined k-mer completeness 97.73%), and mitochondrial and plastid genomes and an Ensembl gene annotation are also provided.

 This is a valuable reference for Apiaceae, all raw data and assemblies are publicly available, and the data themselves appear sound.

 The assembly meets the EBP 6.C.Q40 standard (6.C.Q66), the k-mer completeness benchmark (97.73% for the combined haplotypes) and the chromosome assignment benchmark (96.53%).

 However, haplotype 1 does not meet the BUSCO benchmark stated in Table 5 (S > 90%, D < 5%). For haplotype 1, S = 89.3% (benchmark > 90%) and D = 9.2% (benchmark < 5%), but this is not discussed in the text. The automated benchmarking report scores BUSCO as a pass using a different criterion (> 90% complete BUSCOs), so the publication is currently internally inconsistent. Please either correct the benchmark wording or discuss the elevated duplication. It would help to distinguish genuine gene duplication from residual haplotypic duplication, for example with a Merqury spectra-cn plot for haplotype 1 alone, a purge_dups coverage profile, or the location of the duplicated BUSCOs (tandem, on different chromosomes, or on unplaced scaffolds).

 With 337 scaffolds, a scaffold N50 of 176.38 Mb and a longest scaffold of 237.38 Mb, haplotype 2 is effectively chromosome-scale, and the curation section states that both haplotypes were combined for curation. Why was the haplotype 2 scaffolds not assigned to chromosomes?

 The text states approximately 81× coverage of the haplotype 1 assembly. Dividing the HiFi yield (176.33 Gb) by the haplotype 1 length gives 86.9×, and by the GenomeScope estimate 82.5×. In the blob plot (Figure 6), the chromosomal scaffolds appear to sit at roughly 50× base coverage, by my reading of the plot.

 The assembly (2,028 Mb) represents 85% of the flow cytometry estimate (2,380 Mb) and 95% of the GenomeScope estimate. With 78% repetitive sequence, the missing 350 Mb most likely reflects collapsed tandem repeats, but this is not discussed. Outside the chromosomes, 70.43 Mb remain in 397 scaffolds, and almost all of them (394 scaffolds, 68.8 Mb) have no taxonomic hit. Figure 6 shows a distinct cluster of small scaffolds at 50-52% GC with roughly twice the nuclear coverage, which suggests satellite DNA or rDNA.

Are the datasets clearly presented in a usable and accessible format, and the assembly and annotation available in an appropriate subject-specific repository?

Yes

Are sufficient details of the sequencing and extraction, software used, and materials provided to allow replication by others?

Yes

Are the rationale for sequencing the genome and the species significance clearly described?

Yes

Are the protocols appropriate and is the work technically sound?

Partly

Reviewer Expertise:

plant genome bioinformatics

I confirm that I have read this submission and believe that I have an appropriate level of expertise to confirm that it is of an acceptable scientific standard.

# Reviewer report: Reviewer response for version 1

**Published:** 2026-09-18

**Authors:** Automated Benchmarking

**Affiliations:** 1 Wellcome Sanger Institute, Hinxton, UK

As this article is a Genome Note, Automated Benchmarking has been used to determine whether the reported assembly passes four defined checks established by the Earth BioGenome Project: the raw and assembled data are publicly available, the Earth BioGenome Project three-part metric is met, the k-mer completeness exceeds 90%, and the BUSCO score passes the stated threshold. For Heracleum sphondylium (drHerSpho1), all four checks passed. Data available: pass. ENA study PRJEB80401, raw-read accessions ERR13725938, ERR13725939, ERR13725940, ERR13732194, and ERR13731897, and assembly accessions GCA_965233475.1 and GCA_965233405.1 are public. Earth BioGenome Project three-part metric: pass. Haplotype 1 is reported as 6.C.Q66, meeting the 6.C.Q40 reference standard. k-mer completeness: pass. The combined haplotype assemblies have 97.73% k-mer completeness, exceeding the Earth BioGenome Project target of 90%. BUSCO: pass. Haplotype 1, which is proposed as the reference assembly for this species, has 98.5% complete BUSCOs (89.3% single-copy and 9.2% duplicated). BUSCO software version: 5.7.1; lineage dataset: eudicots_odb10. It meets the plant-specific target of greater than 90% complete BUSCOs. This decision uses values reported in the Genome Note and public accession records.

Reviewer Expertise:

NA

I confirm that I have read this submission and believe that I have an appropriate level of expertise to confirm that it is of an acceptable scientific standard.
